# Characteristics of positive-interaction parenting style among primiparous teenage, optimal age, and advanced age mothers in Canada

**DOI:** 10.1186/s12887-017-0972-z

**Published:** 2018-01-08

**Authors:** Theresa H. M. Kim, Jennifer A. Connolly, Michael Rotondi, Hala Tamim

**Affiliations:** 1 School of Kinesiology and Health Science, York University, 4700 Keele Street, Toronto, ON M3J 1P3 Canada; 2 Department of Psychology, York University, 4700 Keele Street, Toronto, ON M3J 1P3 Canada

**Keywords:** Positive-interaction parenting, Teens, Optimal age women, Advanced age women, Primiparous, Maternal age

## Abstract

**Background:**

Positive-interaction parenting early in childhood is encouraged due to its association with behavioural development later in life. The objective of this study was to examine if the level of positive-interaction parenting style differs among teen, optimal age, and advanced age mothers in Canada, and to identify the characteristics associated with positive-interaction parenting style separately for each age group.

**Methods:**

This was a cross-sectional secondary analysis of the National Longitudinal Survey of Children and Youth. First-time mothers with children 0–23 months were grouped into: teen (15–19 years, *N* = 53,409), optimal age (20–34 years, *N* = 790,960), and advanced age (35 years and older, *N* = 106,536). The outcome was positive-interaction parenting style (Parenting Practices Scale); maternal socio-demographics, health, social, and child characteristics were considered for backward stepwise multiple linear regression modeling, stratified for each of the age groups.

**Results:**

Teen, optimal age, and advanced age mothers reported similar levels of positive- interaction parenting style. Covariates differed across the three age groups. Among optimal age mothers, being an ever-landed immigrant, childcare use, and being devoted to religion were found to decrease positive-interaction parenting style, whereas, higher education was found to increase positive-interaction parenting style. Teen mothers were not found to have any characteristics uniquely associated with positive-interaction parenting. Among advanced age mothers, social support was uniquely associated with an increase in positive-interaction parenting. Very good/excellent health was found to be positively associated with parenting in teens but negatively associated with parenting in advanced age mothers.

**Conclusion:**

Characteristics associated with positive-interaction parenting varied among the three age groups. Findings may have public health implications through information dissemination to first-time mothers, clinicians, researchers, and public health facilities.

## Background

Parenting is a vital component in a child’s life. With appropriate parenting motivation and quality, it may protect children from harm and guide them to healthy physical and emotional wellbeing [1]. It has an immediate effect on behaviour such as aggression, emotion, conduct, and hyperactivity [2–6], and strong associations with improved academic functioning and self-control [7–9]. Since parenting practices have an impact on children’s development and wellbeing, exposure to *positive* parenting early on (when the child is 6-months-old) [10] is encouraged for increased positive behaviour among children [11, 12]. “Positive” or “positive-interaction” parenting [13, 14], is found to be the beneficial to child development [15]. Mothers with positive-interaction parenting display warmth and responsiveness that allows their child to be independent [15, 16]. They are involved with their child’s activities and are engaged in positive reinforcement (e.g., celebrating their child’s accomplishments). They also communicate with their child on matters of conduct behaviour and the differences between good choices and bad choices. Consistently, research has shown that the warmth displayed by the mother positively predicts positive development in the child later in life [17, 18].

The degree and variability that a caregiver displays in positive-interaction parenting may be explained by differences in maternal characteristic such as education, support, and age. In the United States, teen mothers who completed higher education were more likely to be nurturing to their infants compared to those with lower education [19]. Further, support has shown to promote positive parenting practices (i.e., display of acceptance, warmth, and responsiveness) of various cultural backgrounds [20–23]. Parenting style may also differ by differences in age, where lower maternal age has shown to predict harsher and less supportive parenting with toddlers [24–26]. More specifically, teen mothers (19-years-old and under) are reckoned as being less positive, less supportive, and less accessible to their children compared to non-teen mothers [27–30], lacking knowledge of their child’s needs, and lacking emotional maturity to raise a child [29, 31], and are likely to be single parents [32], and therefore, deemed as being at greater risk for suboptimal parenting [27, 30]. Conversely, non-teenaged mothers (20-years-old and older) are regarded as being more positive, more supportive, more able to meet the needs of their child, and being “capable” to parent [27]. Even after accounting for various socio-economic factors, higher maternal age is positively associated with warmth and sensitivity towards their infants [26].

Most literature to date is focused on the maternal parenting styles, with no comparison to advanced age mothers (35-years-old and older) [e.g., 20, 23]. There is a pressing need to examine this group as the number of advanced age mothers in Canada is increasing every year [33]. Furthermore, advanced age mothers are different from teen and optimal age mothers (20–34 years-old), as they usually have higher education and higher income status [34] – both of which are known to influence parenting. Additionally, no research to date has compared the positive-interaction parenting style of teen, optimal age, and advanced age mothers in Canada. This study may help researchers, counselors, and mothers to be acquainted with the significant characteristics associated with positive-interaction parenting, which, in turn may contribute to healthy physical and emotional wellbeing of their children. Therefore, this study aims to examine if the level of positive-interaction parenting style differs among teen, optimal age, and advanced age mothers in Canada, and identifies the characteristics associated with this parenting style separately for each age group.

## Methods

### Sample

This study was a cross-sectional design and was based on the secondary analysis of the National Longitudinal Survey of Children and Youth (NLSCY), sponsored by a joint contribution of Statistics Canada and Human Resources and Skills Development Canada (HRSDC) (formerly Human Resources Development Canada). The NLSCY included data collected from children 0–1 year of age until they turned 4–5 years in all provinces from 1994 to 2008. The survey included information on the child’s biological, social, emotional, and behavioural development, and information on the parent/caregiver’s demographic, social, economic, health, and environment. An interviewer administered the questionnaire using a computer assisted interviewing technique. Participation to the survey was voluntary, and all variables were self-reported. The caregiver (also known as the person most knowledgeable – PMK) provided responses to the questions regarding the child. Rare populations, such as Aboriginals, those institutionalized, residents of the territories, and those who resided in remote areas of Canada were excluded from the study. Full description of the NLSCY is described elsewhere [35]. The total number of women analyzed in this study was 950,905 primiparous Canadian mothers (weighted using population weights) whose child was 0–23 months of age at the time of interview. The sample included 53,409 teen mothers, 790,960 optimal age mothers, and 106,536 advanced age mothers.

### Procedure

The study used data collected from years 2000 to 2008 (cycles 4 to 8). Cycles from earlier than 2000 were excluded because key variables used in the analyses were not consistently measured and changes implemented in cycle 4 were continued through subsequent cycles. The analysis was restricted to: the person most knowledge (PMK) being the biological mother of the child, primiparous, singleton live births, and living with their child (0–23 months-old) at the time of data collection.

### Measures

#### Positive-interaction parenting style

The dependent variable was positive-interaction parenting measured by the *Positive-Interaction Parenting Scale*, analyzed as a continuous variable. The parenting scale in the NLSCY was adapted from Strayhorn and Weidman’s Parenting Practices Scale [36], and measured positive parenting behaviours when the child is 0–23 months [35]. Parents with “positive-interaction” parenting style would report more positive encounters with the child. Items included: “how often do you praise your child”; “how often do you and your child talk or play with each other”; “how often do you and your child laugh together”; “how often do you do something special with your child that he enjoys”; and “how often do you play sports, hobbies, or games with your child”. The total score ranged from 0 to 20, derived from the five items, where a high score indicated a high degree of positive-interaction parenting style. This scale has shown to be valid and reliable (Cronbach’s α = .66–.68) and is described elsewhere [35].

#### Independent variables

The independent variables were: *maternal socio-demographic* (mother’s actual age at childbirth, mother reported ever being a landed immigrant, currently married/living with partner, living in a rural or urban place of residence, mother’s highest level of education completed, self-reported household income in the last 12 months, and mother’s current work status); *maternal health and social* (perceived health status, depression, social support, family functioning, and devotion to religion in the last 12 months); and *child characteristics* (age, sex, use of child care, perceived health status, and temperament). The *data collection year* (survey cycle) was included in the analysis as a covariate. All independent variables were self-reported and were dichotomous/categorical with the exception of age, income, depression, social support, family functioning, and temperament, which were continuous variables or scales. The *Centre for Epidemiological Studies-Depression scale* was a 12-item depression scale, which ranged from 0 to 36, where a high score indicated the presence of depressive symptoms (Cronbach α > .80) [35]. Sample items include: “I could not shake the blues”; and “In the past week, I felt lonely”. The *Social Provisions Scale* was an 8-item social support scale, which ranged from 0 to 24, where a high scored indicated presence of social support (Cronbach α > .89) [35]. Sample items for social support include: “There is someone I trust whom I would turn to for advice if I were having problems”; “I feel part of a group of people who share my attitudes and beliefs”. The *Family Functioning Scale* was a 12-item family dysfunction scale, which ranged from 0 to 36 where a high score indicated presence of family dysfunction (Cronbach’s α = .87) [35]. Sample items for family dysfunction include: “Making decisions is a problem for our family”; “Drinking is a source of tension or disagreement in our family”; and “We don’t get along well together”. Temperament was derived from a question on the overall degree of difficulty the child would present for the average parent, on a scale of 1 to 7 (1 = very easy, 7 = very difficult).

### Statistical analysis

Analyses were stratified for teen mothers (aged 15–19 years), optimal age mothers (aged 20–34 years) and advanced age mothers (aged 35 years and older). Means of positive-interaction parenting style were calculated for each age group. To assess the significant difference of the characteristics across the three age groups, ANOVA was conducted for continuous variables and chi-square test for categorical variables. Unadjusted beta coefficients (β) and 95% confidence intervals (95% CI) were reported for bivariate analysis using simple linear regression for the outcome positive-interaction parenting. Adjusted (Adj) β and 95% CI were reported for the backward stepwise linear regression models, separately for each age group. Population weights to represent the larger Canadian population were applied to estimate sample sizes, unadjusted beta coefficients, and adjusted beta coefficients. To account for the complex sampling design, bootstrapping was performed to calculate standard deviations, standard errors, and 95% confidence intervals. Population weights and bootstrap weights were all created by Statistics Canada and provided with the NLSCY data set. All analyses were performed using Stata (version 13.0 SE). Statistical significance for all analyses was set at α < .05.

### Ethics

Ethics approval was not required as this study was based on secondary data analysis of the NLSCY. Permission to access the NLSCY was obtained through submitting an application to the Social Sciences and Humanities Council of Canada. The NLSCY was accessed at the York Region Research Data Centre in Toronto, Canada.

## Results

The total number of women analyzed in this study was 950,905 primiparous Canadian mothers (weighted using population weights) whose child was 0–23 months of age at the time of interview. The sample included 53,409 teen mothers, 790,960 optimal age mothers, and 106,536 advanced age mothers. Table 1 shows the estimated proportions of characteristics among all primiparous mothers with children 0–23 months old. Teen, optimal age, and advanced age mothers reported similar levels of positive-interaction parenting style as shown in Table 1 (*p = .*270). Proportions in marital status across age groups significantly differed (*p* < .001); only 52.2% of teen mothers reported being married or with a partner compared to optimal age and advanced age mothers whereby majority (> 90%) reported having a partner. Only 9.7% of teen mothers were ever immigrants whereas, 17.6% of optimal age, and 30.0% of advanced age mothers were immigrants (*p* < .001). A greater proportion of teen mothers reported obtaining a high school education or less (69.8%) whereas a greater proportion (> 60%) of optimal and advanced age mothers reported obtaining postsecondary or partial university, with a larger proportion of advanced age mothers obtaining a bachelor degree or higher compared to optimal age mothers (*p* < .001). Teen mothers scored higher on the depression scale (5.62) than optimal (3.72) and advanced age mothers (3.89) (*p* < .001). Teen mothers reported the highest family dysfunction and lowest social support compared to optimal age and advanced age mothers (*p* < .001 and *p =* .005 respectively).

**Table 1:** Characteristics of primiparous teenage, optimal age, and advanced age mothers living with children 0–23 month-old

	Teenage Mothers *N* = 53,409	Optimal Age Mothers *N* = 790,960	Advanced Age Mothers *N* = 106,536	
	%	%	%	*p*-value*^b^
Positive-Interaction Parenting				
Mean^a^ (SE)^b^	18.14 (.12)	18.27 (.04)	18.40 (.11)	.270
*Maternal Socio-Demographic Characteristics*				
Mother’s Age at Childbirth				
Mean^a^ (SE)^b^	18.19 (.07)	27.16 (.08)	37.27 (.11)	**< .001**
Immigration to Canada				
No Yes	90.3 9.7	82.4 17.6	70.0 30.0	**< .001**
Married/With Partner				
No Yes	47.8 52.2	9.7 90.3	7.7 92.3	**< .001**
Place of Residence				
Rural Urban	12.2 87.8	9.8 90.2	5.9 94.1	**0.009**
Level of Education				
High school or less Postsec/Part University Bachelor or higher	69.8 30.2 N/A	16.4 65.1 18.5	7.5 64.2 28.3	**< .001**
Household Income ($1000’s)				
Mean^a^ (SE)^b^	32.46 (1.57)	70.59 (1.28)	89.86 (3.62)	**< .001**
Work Status				
No work in past year Worked part-time last year Worked full-time last year	47.7 28.8 23.5	31.7 17.7 50.6	27.4 16.3 56.3	**< .001**
*Maternal Health and Social Characteristics*				
Perceived Health Status				
Good/Fair/Poor Health Very good/Excellent Health	35.8 64.2	22.1 77.9	24.7 75.3	**< .001**
Depression				
Mean^a^ (SE)^b^	5.62 (.32)	3.72 (.08)	3.89 (.28)	**< .001**
Family Functioning				
Mean^a^ (SE)^b^	10.32 (.31)	8.15 (.10)	8.79 (.33)	**< .001**
Social Support				
Mean^a^ (SE)^b^	17.88 (.21)	19.11 (.08)	18.92 (.22)	**.005**
Devotion to Religion				
Never attended Partially/regularly attended	50.4 49.6	43.5 56.5	46.6 53.4	.125
*Child Characteristics*				
Age of Child (Months)				
Mean^a^ (SE)^b^	13.56 (.37)	13.04 (.10)	13.53 (.27)	.254
Sex of Child				
Male Female	52.0 48.0	52.9 47.1	51.4 48.6	.858
Use of Childcare				
No Yes	68.2 31.8	73.3 26.7	72.2 27.8	.285
Health Status of Child				
Good/Fair/Poor Health Improved Health	11.8 88.2	6.4 93.6	5.2 94.8	**.009**
Temperament				
Mean^a^ (SE)^b^	2.72 (.51)	2.57 (.14)	2.66 (.35)	.112
*Survey Data*				
Data Collection Year				
Cycle 4 (2000–2001) Cycle 5 (2002–2003) Cycle 6 (2004–2005) Cycle 7 (2006–2007) Cycle 8 (2008–2009)	19.4 21.0 22.5 18.7 18.4	16.0 17.0 19.6 24.5 22.9	14.8 20.4 18.3 20.4 26.1	.102

Values represent column percentages estimated using population weights, unless otherwise indicated

*p-values < .05 denote significance, and refer to between-subject groups; bolded *p*-values denote significance

^a^Sample size and means are estimated using population weights

^b^Standard error values and between group *p*-values were calculated using bootstrap weights

Table 2 shows the results from simple linear regression and stepwise multivariable linear regression, with the unadjusted and adjusted beta coefficients of positive-interaction parenting and related characteristics for each group. At the bivariate level, no significant difference was found for positive interaction parenting across the three age groups, however, in an overall adjusted model and when several interaction terms were added, maternal age along with a maternal age interaction term were found to be significant, providing justification to stratify the analysis by maternal age. Among optimal age mothers, positive-interaction parenting was decreased with ever-landed immigrant status Adj β = −.42, 95%CI -.71, −.14), and significantly increased with increasing education, after adjusting for other variables. Furthermore, depression (Adj β = −.03, 95%CI -.05, −.01), family functioning (Adj β = −.05, 95%CI -.06, −.03), and devotion to religion (Adj β = −.19, 95%CI -.33, −.04) were negatively associated with positive-interaction parenting in optimal age mothers after adjustment. Among optimal age mothers, age of the child and use of childcare were negatively significant with positive-interaction parenting. Among teen mothers, positive-interaction parenting significantly increased with very good/excellent health and significantly decreased with poor family functioning (Adj β = −.05, 95%CI -.10, −.01) after adjustment of variables. Among advanced age mothers, parenting significantly decreased with age of the child, very good/excellent health, and depression, and significantly increased with social support (Adj β = .08, 95%CI .03, .13), after adjusting for other variables.

**Table 2:** Estimated unadjusted and adjusted beta coefficients of *positive-interaction parenting* and related characteristics among primiparous mothers with children 0–23 month-old

	Teen Mothers *N* = 49,050		Optimal Age Mothers *N* = 748,683		Advanced Age Mothers *N* = 100,137	
	Unadjusted β^a^ (95%CI)^b^	Adjusted β^a^ (95%CI)^b^	Unadjusted β^a^ (95%CI)^b^	Adjusted β^a^ (95%CI)^b^	Unadjusted β^a^ (95%CI)^b^	Adjusted β^a^ (95%CI)^b^
*Maternal Socio-Demographic Characteristics*						
Mother’s Age at Childbirth	.11 (−.10, .32)		.02 (−.001, .04)		−.01 (−.12, .10)	
Ever Immigrants of Canada^1^	.15 (−.92, 1.21)		**−.53 (−.83, −.23)**	**−.42 (−.71, −.14)**	−.30 (−.87, .28)	
Married/With Partner^2^	.24 (−0.24, .71)		.33 (−.02, .68)		−.17 (−.69, .35)	
Resides in Urban Population^3^	−.06 (−.70, .57)		−.09 (−.26, .08)		−.29 (−.83, .25)	
Postsecondary/Part University^4^ Bachelor Degree or higher^4^	.13 (−.40, .65) N/A		**.35 (.10, .61)** **.48 (.19, .77)**	**.27 (.02, .53)** **.39 (.08, .71)**	−.10 (−.75, .55) −.14 (−.83, .56)	
Household Income	.01 (−.003, .02)		**.002 (.001, .004)**		.001 (−.002, .004)	
Working Part-time^5^ Working Full-time^5^	.19 (−.36, .75) −.22 (−.88, .45)	.19 (−.38, .77) −.31 (−.96, .34)	.19 (−.02, .40) −.02 (−.22, .18)	.15 (−.05, .36) −.09 (−.29, .10)	−.39 (−1.07, .29) −.24 (−.79, .31)	
*Maternal Health and Social Characteristics*						
Very good/Excellent Health^6^	**.86 (.38, 1.34)**	**.77 (.22, 1.31)**	.15 (−.02, .32)		**−.41 (−.78, −.03)**	**−.62 (−1.04, −.20)**
Depression	−.04 (−.09, .01)		**−.05 (−.07, −.03)**	**−.03 (−.05, −.01)**	**−.07 (−.13, −.01)**	**−.07 (−.13, −.01)**
Family Functioning	**−.06 (−.10, −.01)**	**−.05 (−.10, −.01)**	**−.06 (−.07, −.04)**	**−.05 (−.06, −.03)**	**−.05 (−.10, −.01)**	
Social Support	.03 (−.05, .11)		**.06 (.04, .08)**		**.08 (.03, .14)**	**.08 (.03, .13)**
Devoted to Religion^8^	.27 (−.21, .75)		**−.20 (−.35, −.04)**	**−.19 (−.33, −.04)**	−.28 (−.69, .13)	
*Child Characteristics*						
Age of Child (months)	−.05 (−.10, .01)		**−.04 (−.05, −.02)**	**−.03 (−.05, −.01)**	**−.06 (−.09, −.02)**	**−.05 (−.09, −.01)**
Female Child^9^	**−.46 (−.92, −.01)**		−.02 (−.18, .14)		−.34 (−.76, .09)	
Used Child Care^7^	−.04 (−.56, .48)		**−.26 (−.42, −.09)**	**−.29 (−.48, −.10)**	−.30 (−.71, .11)	
Improved Health^6^	.34 (−.66, 1.33)		**.44 (.08, .79)**		.59 (−.54, 1.72)	
Overall Temperament	.01 (−.14, .17)		.01 (−.0003, .01)		−.002 (−.09, .08)	−.003 (−.08, .07)
*Survey Data*						
Cycle 5 (2002–2003)^10^ Cycle 6 (2004–2005)^10^ Cycle 7 (2006–2007)^10^ Cycle 8 (2008–2009)^10^	.24 (−.31, .80) −.29 (−.94, .36) −.30 (−1.07, .46) .29 (−0.18, .76)		.28 (.11, .46) −.09 (−.28, .10) −.13 (−.35, .10) .12 (−.05, .30)	**.35 (.12, .59)** .05 (−.20, .30) −.12 (−.43, .19) .05 (−.20, .31)	.23 (−.27, .73) .15 (−.30, .60) −.18 (−.87, .51) −.27 (−.71, .17)	

Values represent unstandardized beta coefficients and 95% confidence intervals in parentheses estimated using population weights. Bolded values in the table denote significance

^a^Sample size and beta coefficients were estimated using population weights

^b^95% Confidence Interval (CI) and standard error values were calculated using bootstrap weights

^1^Reference category: Non-immigrants- Canadian born; ^2^Reference: non-married; ^3^Reference: rural population; ^4^Reference: High school or less; ^5^Reference: Not working; ^6^Reference: Good/Fair/Poor perceived health status; ^7^Reference: No child care used; ^8^Reference: No devotion to religion; ^9^Reference: Male ^10^Reference: Cycle 4 (2000–2001)

To estimate effect sizes, coefficients were re-scaled to standardized regression coefficients to provide some indication of the magnitude of these effects. In teenage mothers, small effect sizes were observed for very good/excellent health (0.20), and family functioning (0.13). In optimal age mothers, among variables that were found to be significantly associated with positive-interaction parenting style, all indicated a standardized regression coefficient of less than 20. In advanced age mothers, all variables that were found to be significant indicated a standardized regression coefficient of less than 20.

## Discussion

This study identified and compared the characteristics of positive-interaction parenting style among teen, optimal age, and advanced age mothers using a Canadian-wide dataset. There was no significant difference in the frequency of positive-interaction parenting style across the three age groups however, at the multivariable model, maternal age was found to be significant when several interaction terms were added in the model. Although a lack of statistical significance does not guarantee no effect, the significant predictors retained in each of the models for age of the mother showed that associated characteristics differed*.* Among optimal age mothers, positive-interaction parenting significantly increased with higher education, and decreased with ever-immigrants, depression, family dysfunction, devotion to religion, age of the child, and childcare use. Among teens, positive-interaction parenting significantly increased with very good/excellent health, and decreased with family functioning. Among advanced age mothers, positive-interaction parenting significantly increased with social support, and decreased with depression, very good/excellent health, and older children. These findings will be relevant to professionals working in the area of counseling, family medicine, nursing, and public health, allowing them to identify the unique and overlapping characteristics that significantly predict positive-interaction parenting style in the three age groups of mothers.

Teen, optimal age, and advanced age mothers reported similar levels of positive-interaction parenting. This finding is inconsistent with previous studies whereby teen mothers were found to be harsher and display less positive parenting styles than older mothers who were generally more positive and warm toward their child [24, 26, 27, 30]. This was based on the premise that teen mothers lack emotional maturity and knowledge about the child [29, 31], thereby not being able to positively interact with their children. A probable reason for the inconsistency with other studies is the difference in target population. This study assessed parenting when the child was 0–23 months old, whereas others examined parenting styles when the child was older. All three groups may have similarly displayed high levels of positive-interaction parenting, as children under 2 years of age have not yet reached the “age of understanding” [37], at which point, more of a disciplinary action may be imposed. Therefore, further investigation on parenting styles among the three age groups with older children in Canada is warranted.

No characteristic was found to be commonly significant across all three age groups. The characteristics uniquely associated with optimal age mothers were ever-landed immigrants, education, childcare use, and devotion to religion. Optimal age mothers who were ever-landed immigrants reported less positive-interaction parenting compared to non-immigrants. Similarly, another study found that immigrant mothers showed a harsher and punitive type of parenting compared to non-immigrants in the United Kingdom and Turkey [38]. It is understood that immigrants try to retain their traditional family values that may allow for more restrictive behaviour towards their children to protect them from “perceived” risk in an unfamiliar country [39]. In contrast, a study in the United States found that immigrant mothers reported warmer and intimate parenting styles compared to non-immigrants [20, 40, 41]. The inconsistencies found in these studies may be due to the cultural differences (i.e., Turkey versus United States), and different parenting scales used for assessment. Similarly, in other studies, positive-interaction parenting significantly increased with increasing education in optimal age mothers [20, 42, 43]. Mothers with higher education (graduate or professional degrees) were more likely to display positive-interaction parenting, as they were likely to have a better understanding of the importance of positive-interaction parenting on the social and cognitive development of children [20, 42, 43]. On the other hand, positive-interaction parenting was associated with a decrease in childcare use. However, due to reverse causality and the lack of information on duration and quality of childcare use, further investigation is warranted to explore this relationship. Although some studies show that devotion to religion is associated with physical affection and praising of their children [44, 45], our results show that being devoted to religion decreased positive-interaction parenting. In certain contexts, those who display stricter parenting hold stronger religious views about disciplinary actions such as setting limits and using corporal punishment for unacceptable behaviour [44, 45].

Teen mothers were not found to have any characteristics uniquely associated with positive-interaction parenting. However, social support was uniquely associated with parenting among advanced age mothers. In general, social support promotes positive-interaction parenting by enhancing parents’ psychological functioning [46, 47]. Mothers with greater social support are more likely to display positive interactions with their children [47], and are likely to report better parent-child involvement and communication [46]. Similar results were produced in the United Kingdom where younger mothers exhibited lower rates of positive-interaction parenting compared to older mothers [48].

Positive-interaction parenting style significantly decreased with family dysfunction in teen and optimal age mothers. Similarly, poor parenting was found to be associated with family dysfunction where it places children at risk for illnesses, substance misuse, and juvenile crime [1]. Very good/excellent health was found to be positively associated with parenting in teens but negatively associated with parenting in advanced age mothers. Although the relationship of health and positive-interaction parenting in teens is well supported by another study [49], the reason why this relationship is different for advanced age mothers warrants further investigation.

Optimal age and advanced age mothers were found to have positive-interaction parenting styles significantly decrease with depression. Previous studies have also shown a link between maternal depression and lower positive-interaction parenting [42, 46, 50]. Depressed mothers are likely to be less engaged and show negative affect towards their children compared to non-depressed individuals [46, 51]. Although it has been shown that mothers with older toddlers display more positive maternal behaviour [52], our study showed that positive-interaction parenting decreased with older children. However, as children in our study have not yet reached the “age of understanding” [37], further investigation among children older than 23 months is warranted.

### Strengths and limitations

The results should be cautiously interpreted, as limitations are present. There is potential for reverse causality between the dependent and independent variables due to the cross-sectional nature of the study. Furthermore, information bias may be present as all variables were self-reported however, variables such as the positive-interaction parenting, social support, family functioning, and depression are validated measures that have been effectively used in other studies. Although our study explored numerous variables, other parenting-influenced characteristics such as the mother’s mental health, social skills, and attendance of parenting classes were not captured in the NLSCY, and thus not part of our analyses. The data used in this study was collected from 2000 to 2008; therefore, it may not represent the current population. However, the survey weights applied to the data set allowed the sample to be generalizable at the population level that includes a standardized parenting scale and covers a comprehensive range of topics including health, behaviour, and social environment of the mother and her child.

## Conclusion

Our study is novel in comparing the characteristics of positive-interaction parenting style among three age groups of mothers in Canada using a nation-wide dataset. All mothers reported similar levels of positive-interaction parenting however, unique characteristics associated with parenting were present. There were no common variables significant with parenting across the three groups however; mother’s perceived health was the most thought-provoking finding in our study. Very good/excellent health predicted positive-interaction parenting differently for teen and advanced age mothers, advising readers to be mindful that maternal health may be an important contributor to parenting, albeit its effect size of 0.20 noted in our results. The key to better understanding this relationship may lie within conducting further research with advanced age mothers as this area is still in its infancy. Our findings may have strong public health implications through information dissemination to first-time mothers, clinicians, researchers, and public health facilities. Targeting these audiences with information on how perceived health may impact parenting differently based on maternal age may be one strategy to increase awareness. Further, the implications of this study may help the academic community to foster partnerships with policy-making bodies to inform evidence-based recommendations specific to health status and positive-interaction parenting.

## Abbreviations

Adj: Adjusted beta coefficient
ANOVA: Analysis of variance
HRSDC: Human Resources and Skills Development Canada
NLSCY: National Longitudinal Survey of Children and Youth
PMK: Person most knowledgeable

## References

[1] Hoghughi M (1998). The importance of parenting in child health. Br Med J.

[2] Benzies K, Keown L-A, Magill-Evans J (2009). Immediate and sustained effects of parenting on physical aggression. Can J Psychiatr.

[3] Cowan PA, Cowan CP (2002). Interventions as tests of family systems theories: marital and family relationships in children’s development and psychopathology. Dev Psychopathol.

[4] Spoth RL, Redmond C, Shin C (2000). Reducing adolescent’s aggressive and hostile behaviors: randomized trial effects of a brief family intervention 4 years past baseline. Arch Pediatr Adolesc Med.

[5] Azevedo FA, Seabra-Santos MJ, Gaspar MF, Homem T (2014). A parent-based intervention programme involving preschoolers with AD/HD behaviours: are children’s and mothers’ effects sustained over time?. Eur Child Adolesc Psychiatry.

[6] Brotman LM, O’Neal CR, Huang KY, Gouley KK, Rosenfelt A, Shrout PE (2009). An experimental test of parenting practices as a mediator of early childhood physical aggression. J Child Psychol Psychiatry.

[7] Bradley B, Davis TA, Kaye J, Wingo A, Kent M, Davis MC, Reich JW (2013). Developmental social factors as promoters of resilience in childhood and adolescence. The resilience handbook: approaches to stress and trauma.

[8] Cheung CS, McBride-Chang C (2008). Relations of perceived maternal parenting style, practices, and learning motivation to academic competence in Chinese children. Merrill-Palmer Q.

[9] VanVoorhis FL (2011). Costs and benefits of family involvement in homework. Journal of Advanced Academics.

[10] Glascoe FP, Leew S (2010). Parenting behaviors, perceptions, and psychosocial risk: impacts on young children’s development. Pediatrics.

[11] Chronis AM, Lahey BB, Pelham WE, Williams SH, Baumann BL, Kipp H, Jones HA, Rathouz PJ (2007). Maternal depression and early positive parenting predict future conduct problems in young children with attention deficit/hyperactivity disorder. Dev Psychol.

[12] Dallaire DH, Pineda AQ, Cole DA, Ciesla JA, Jacquez F, Lagrange B, Bruce AE (2006). Relation of positive and negative parenting to children’s depressive symptoms. J Clin Child Adolesc Psychol.

[13] Browne DT, Odueyungbo A, Thabane L, Byrne C, Smart LA (2010). Parenting-by-gender interactions in child psychopathology: attempting to address inconsistencies with a Canadian national database. Child and Adolescent Psychiatry and Mental Health.

[14] Kakinami L, Barnett TA, Seguin L, Paradis G (2015). Parenting style and obesity risk in children. Prev Med.

[15] Baumrind D (1991). The influence of parenting style on adolescent competence and substance use. J Early Adolesc.

[16] Baumrind D (1966). Effects of authoritative parental control on child behavior. Child Dev.

[17] Schofield TJ, Conger RD, Donnellan MB, Jochem R, Widaman KF, Conger KJ (2012). Parent personality and positive parenting as predictors of positive adolescent personality development over time. Merrill Palmer Q.

[18] Garcia F, Garcia E (2009). Is always authoritative the optimum parenting style? Evidence from Spanish families. Adolescence.

[19] Hess CR, Papas MA, Black MM (2002). Resilience among African American adolescent mothers: predictors of positive parenting in early infancy. J Pediatr Psychol.

[20] Cheah CSL, Leung CYY, Tahseen M, Schultz D (2009). Authoritative parenting among immigrant Chinese mothers of preschoolers. J Fam Psychol.

[21] Serrano-Villar M, Huang K-Y, Calzada EJ. Social support, parenting, and social emotional development in young Mexican and Dominican American children. Child Psychiatry Hum Dev. 2016. 10.1007/s10578-016-0685-9. Epub ahead of print. 10.1007/s10578-016-0685-9 27696243

[22] Ceballo R, McLoyd VC (2002). Social support and parenting in poor, dangerous neighborhoods. Child Dev.

[23] Izzo C, Weiss L, Shanahan T, Rodriguez-Brown F (2000). Parental self-efficacy and social support as predictors of parenting practices and children’s socioemotional adjustment in Mexican immigrant families. J Prev Interv Commun.

[24] Trentacosta CJ, Neppl TK, Donnellan MB, Shaw DS, Conger RD (2010). Adolescent personality as a prospective predictor of parenting: an interactionist perspective. J Fam Psychol.

[25] Scaramella LV, Neppl TK, Ontai LL, Conger RD (2008). Consequences of socioeconomic disadvantage across three generations: parenting behavior and child externalizing problems. J Fam Psychol.

[26] Bornstein MH, Putnick DL, Suwalsky JTD, Gini M (2006). Maternal chronological age, prenatal and perinatal history, social support, and parenting of infants. Child Dev.

[27] Reis J (1989). A comparison of young teenage, older teenage, and adult mothers on determinants of parenting. The Journal of Psychology.

[28] Trad P (1995). Mental health of adolescent mothers. Journal of American Academy of Child and Adolescent Psychiatry.

[29] Phipps-Yonas S (1980). Teenage pregnancy and motherhood: a review of literature. Amer J Orthopsychiat.

[30] Lewin A, Mitchell SJ, Ronzio CR (2013). Developmental differences in parenting behavior: comparing adolescent, emerging adult, and adult mothers. Merrill-Palmer Q.

[31] Whiteside-Mansell L, Pope S, Bradley P (1996). Patterns of parenting behavior in young mothers. Fam Relat.

[32] Al-Sahab B, Heifetz M, Tamim H, Bohr Y, Connolly J (2012). Prevalence and characteristics of teen motherhood in Canada. Matern Child Health J.

[33] Statistics Canada. (2015). Table 102–4507 – live births, by age and marital status of mother, Canada, annual. 2015. http://www5.statcan.gc.ca/cansim/a26?lang=eng&retrLang=eng&id=1024507&tabMode=dataTable&srchLan=-1&p1=-1&p2=9#F1. Accessed 30 Jan 2016.

[34] Bayrampour H, Heaman M (2011). Comparison of demographic and obstetric characteristics of Canadian primiparous women of advanced maternal age and younger age. J Obstet Gynaecol Can.

[35] Statistics Canada and Human Resources and Skills Development Canada (HRSDC) (2009). National Longitudinal Survey of children and youth. User’s handbook and microdata guide.

[36] Strayhorn JM, Weidman CS (1988). A parent practices scale and its relation to parent and child mental health. Journal of American Child and Adolescent Psychiatry.

[37] Ho DYF (1989). Continuity and variation in Chinese patterns of socialization. J Marriage Fam.

[38] Daglar M, Melhuish E, Barnes J (2011). Parenting and preschool child behaviour among Turkish immigrant, migrant and non-migrant families. European Journal of Developmental Psychology.

[39] Querido JG, Warner TD, Eyberg SM (2002). Parenting styles and child behaviour in African-American families of pre-school children. Journal of Clinical Child Psychology.

[40] Jose PE, Huntsinger CS, Huntsinger PR, Liaw FR (2000). Parental values and practices relevant to young children’s social development in Taiwan and the United States. J Cross-Cult Psychol.

[41] Chao RK (1995). Chinese and European American cultural models of the self reflected in mothers’ childrearing beliefs. Ethos.

[42] Azad G, Blacher J, Marcoulides G (2014). Longitudinal models of soci-economic status: impact on positive parenting behaviors. Internal Journal of Behavioural Development.

[43] Belsky J, Bell B, Bradley RH, Stallard N, Lynette S, Steward-Brown SL (2006). Socioeconomic risk, parenting during the preschool years and child health age 6 years. Eur J Pub Health.

[44] Mahoney A (2010). Religion in families, 1999-2009: a relational spirituality framework. J Marriage Fam.

[45] Mahoney A, Pargament KI, Tarakeshwar N, Swank AB (2001). Religion in the home in the 1980s and 1990s: a meta-analytic review and conceptual analysis of links between religion, marriage, and parenting. J Fam Psychol.

[46] Lee C-YS, Anderson JR, Horowitz JL, August GJ (2009). Family income and parenting: the role of parental depression and social support. Fam Relat.

[47] Jennings KD, Stagg V, Connors RE (1991). Social networks and mothers’ interactions with their preschool children. Child Dev.

[48] Thomson RM, Allely CS, Purves D, Puckering C, McConnachie A, Johnson PCD, Golding J, Gillberg C, Wilson P (2014). Predictors of positive and negative parenting behaviours: evidence from the ALSPAC cohort. BMC Pediatr.

[49] Torquati JC (2002). Personal and social resources as predictors of parenting in homeless families. J Fam Issues.

[50] Parke RD, Coltrane S, Duffy S, Buriel R, Dennis J, Powers J, Widaman KF (2004). Economic stress, parenting, and child adjustment in Mexican American and European American families. Child Dev.

[51] Lovejoy MC, Graczyk PA, O’Hare E, Neuman G (2000). Maternal depression and parenting behavior. A meta-analytic review. Clin Psychol Rev.

[52] Nitz K, Ketterlinus RD, Brandt LJ (1995). The role of stress, social support, and family environment in adolescent mothers’ parenting. J Adolesc Res.

